# Combining Users' Cognition Noise with Interactive Genetic Algorithms and Trapezoidal Fuzzy Numbers for Product Color Design

**DOI:** 10.1155/2019/1019749

**Published:** 2019-08-01

**Authors:** Yan-pu Yang, Xing Tian

**Affiliations:** School of Construction Machinery, Chang'an University, Xi'an 710064, China

## Abstract

Product color plays a vital role in shaping brand style and affecting users' purchase decision. However, users' preferences about product color design schemes may vary due to their cognition differences. Although considering users' perception of product color has been widely performed by industrial designers, it is not effective to support this activity. In order to provide users with plentiful product color solutions as well as embody users' preference into product design process, involving users in interactive genetic algorithms (IGAs) is an effectual way to find optimum solutions. Nevertheless, cognition difference and uncertainty among users may lead to various understanding in line with IGA progressing. To address this issue, this study presents an advanced IGA by combining users' cognition noise which includes cognition phase, intermediate phase, and fatigue phase. Trapezoidal fuzzy numbers are employed to represent uncertainty of users' evaluations. An algorithm is designed to find key parameters through similarity calculation between RGB value and their area proportion of two individuals and users' judgment. The interactive product color design process is put forward with an instance by comparing with an ordinary IGA. Results show that (1) knowledge background will significantly affect users' cognition about product colors and (2) the proposed method is helpful to improve convergence speed and evolution efficiency with convergence increasing from 67.5% to 82.5% and overall average evolutionary generations decreasing from 18.15 to 15.825. It is promising that the proposed method can help reduce users' cognition noise, promote convergence, and improve evolution efficiency of interactive product color design.

## 1. Introduction

As an essential component of a vision system, color can trigger complex aesthetic sensations and psychological reactions and impact on the cognition and emotions of people [1]. As an important marketing communication tool, color carries abundant visual, symbolic, and associative information about products [2]. Since color is of great importance in visualizing appearance of products, manipulating product color has become an important way to touch off consumers' emotional experience, attract consumers' attention, and convince them to buy a product [2, 3]. A proper selection of product color can not only code visual information and communicate a brand's positioning but also help build product style and induce different feelings [4, 5]. Consumers' feelings about a product reflect their psychological preferences and are determined by their inner perceptions [6]. In this light, how to integrate consumers' perceptions into product color design process effectively becomes a critical issue for successful product development.

Product color design refers to selecting appropriate colors in order to convey the desired emotion to consumers. Due to the shortened product life cycle and the diversified product demand, it is becoming crucial for enterprises to realize fast innovation, which makes computer-aided techniques and intelligent algorithms rise and be widely used to adapt to consumers' expectations. There have been several approaches to assist and support intelligent product color design, including using genetic algorithm (GA) for a near-optimal color combination design for multicolored products [7], employing particle swarm optimization (PSO) to find product color solutions that fit with consumers' multiemotions [8], integrating factor analysis, fuzzy analytic hierarchy process, and image compositing technique to analyze consumers' subjective perceptions for customized product color design [9], combining grey theory and GA to search for color combinations that meet the specified product color emotions and achieved a high degree of color harmony [10] and developing computer-aided color planning system to obtain optimized natural colors of an image and transfer them into product color design [11]. To reflect the designer's subjective experience, the interactive genetic algorithm (IGA) is created to establish a creative and interactive evolutionary system that a designer can participate and interact to explore novel design schemes [12]. The relevant research results have been successfully applied in various fields of color design, such as web page [13], arm-type aerial work platform [14], clothes [15], automotive exterior [16], and electronic door lock [17].

However, cognitive dissonance often occurs because consumers do not have the systematic training as designers do to colors, which will lead to inconsistent perceptions between consumers and designers [9]. Cognitive dissonance arises when there are discrepancy and inconsistency between cognitions [18], and people are more comfortable with consistency than inconsistency [19]. As design often is interdisciplinary in practice and members of a team may have different knowledge and expertise, how to reduce cognitive dissonance in design process becomes a challenge. An effective way presented by Goel and Wiltgen [20] is to employ analogies as a mechanism for reducing cognitive dissonance in interdisciplinary design teams. As a reasoning process in creative design, analogies can conduce to reduction of individual differences, similar to the knowledge sharing method [21]. In the industrial design field, a powerful approach is Kansei engineering, which can help designer link consumers' emotional response to design properties of a product [22–24]. For computational and intelligent solving, the effective method is IGA, which involves human as evaluators to make evaluations and selections, and get the fitness value in an evolutionary process instead of making the fitness function in classical genetic algorithms [25, 26]. IGA is conducive to capture consumers' aesthetic intention and percept users' emotion or preference [27], and has been widely used in color design of various products, such as motorcycle [12], car console [28, 29], software robot [30], etc. Nevertheless, users' fatigue and cognitive dissonance are ubiquitous and will gradually arise with the evolution process of IGAs, which can be defined as fitness noise and will influence the performance of interactive evolutionary computation (IEC) [31]. The former can be caused by a lot of repetitive work, tedious operation and visual weariness, and the latter may be attributed to the user's knowledge and experience discrepancy. They have constituted the issues and obstacles to the application of IGAs. To solve these problems, many researchers have studied and put forward several practical methods, such as using multistage IGAs to divide population into several stages for lessening users' population cognition burden [28], adopting a fuzzy number described with a Gaussian membership function to express an individual's fitness [32], employing preference surrogate model to achieve fitness estimation and information extraction in the process of IEC [33–35], etc.

As the intelligent evolutionary method is based on users' preference and selection, the fitness of each individual in evolution process is gained by users' subjective evaluation, which may be affected by users' experiences, cognitive disparities, or fatigue, leading to unobjective evaluation [28]. In other words, the fitness given by users always mixes with noise and is imprecise in the evolution process, and the evolutionary consequences cannot reflect consumers' preferences accurately and ultimately affect the accuracy and validity of final design decisions. Although several researches have proposed methods to address these issues, the effects of these problems cannot be completely eliminated. It is still interesting and worthy of further research.

This study presents an IGA method that considers the influence of cognition noise, including consumers' cognition familiarity, and fatigue and employs trapezoidal fuzzy numbers to represent the uncertainty of users' judgment instead of precise values. To do so, a cognition noise model is proposed by considering three phases: cognitive phase, intermediate phase, and fatigue phase. By considering cognitive noise and introducing trapezoidal fuzzy numbers, the IGA method can reduce the influence of subjectivity of consumers' evaluation. To validate the proposed method, the IGA method is adopted for designing a handheld detector.

The remainder of the paper is organized as follows: Section 2 introduces the methods for interactive product color design, including cognition noise model by combining users' cognition familiarity and fatigue, a solving algorithm through similarity measurement of the individual's color and evaluation with trapezoidal fuzzy numbers, and an interactive product color design process. Then, a numerical example is provided to illustrate the detailed implementation of the proposed method in Section 3. Finally, we summarize and highlight the contribution of this paper.

## 2. Methods

IGA is an optimization method that connects a computer system and human being to jointly accomplish a task [36]. It provides a framework for interaction between humans and computers where computers use GAs to explore possible solutions and converge them to adapt objectives and constraints, and humans evaluate and provide feedback on individuals in the search process. Because of the characteristics that the fitness values of individuals are computed by users' assigned preference rank rather than numerical calculation, IGA is effective to solve problems that indexing optimization of the implicit performance cannot be directly calculated by a function [37]. Since users participate in the IGA process, it is inevitable that users' cognition about individuals will change and users' fatigue will emerge as the population involves. Several approaches have been explored to help reduce users' cognition burden and alleviate their fatigue, including dividing interactive design process into several stages to lower population complex in the initial stage [28], incorporating a case-based machine learning system to learn and predict user's assessment [38], and training artificial neural network to automatically define an iterative fitness function [39]. These researches provide feasible methods to decrease human fatigue to a certain extent. As human fatigue cannot be completely eliminated, it is necessary to solve this dilemma by removing instead of only reducing the impact on evolution in product color design process through appropriate algorithm design. To do so, we build a users' cognition noise model and develop a solving algorithm for interactive product color design.

### 2.1. Users' Cognition Noise Model

In an IGA process for product color design, the fitness values of individuals are likely to change with users' cognitive level, which embodies in two aspects.

1. In the initial stage of an IGA, the users may not be familiar with product color schemes, and it is not easy to obtain precise cognition about individuals from users, leading to the evaluation results carrying a greater randomness. As interactive evolution progresses, the users can get much clearer cognition about individuals, and the users' cognition is advancing towards more comprehension, which can be used as a stable evaluation standard. Although there is a certain degree of randomness at this time, the random noise is relatively small. According to the above analysis, we might describe the problem as follows: Set the cognition threshold as *N*_c_, that is, the users are completely familiar with the individuals after evaluating *N*_c_ product color schemes. When the number of individuals exceeds *N*_c_, the users' evaluation can be identified with no noise. The simpler the product color schemes are, the smaller the *N*_c_ will be, and vice versa.
2. After the user's cognition getting more comprehension, when the number of product color schemes that have been evaluated reaches a certain threshold, the user might get fatigued. At this time, the given fitness value cannot accurately reflect the user's preference and the quality of color schemes. Set the fatigue threshold as *N*_f_, that is, users begin to get fatigue after evaluating *N*_f_ product color schemes.

Assume that the evaluation process has reached generation *t* and *i* + 1 product color schemes have been assessed, then the number of evaluated individuals can be depicted as *N*_e_ = (*t* − 1) · *N* + *i*, where *N* represents the number of individuals per generation. When *N*_e_ < *N*_c_, the users' cognition about product color schemes is proportional to *N*_e_. That means as the numbers of evaluated individuals increase, the users' familiarity will largely improve. When *N*_e_ ≥ *N*_f_ ≥ *N*_c_, even if the users are already familiar with the individuals, they would get fatigued, and as a result, fatigue noise will affect the evolution process. For ease of processing, this study assumes *N*_f_ ≥ *N*_c_.

**Figure 1:**
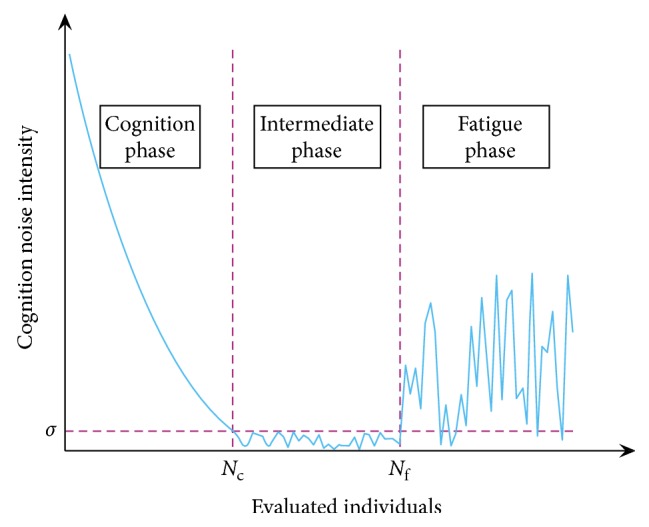
The curve of cognition noise intensity varies with the number of evaluated individuals.

Based on the above analysis, the users' cognition noise model is constructed as follows:

(1) $$\begin{array}{c} \delta \left(N_{\text{e}}\right)=\left\{\begin{array}{cc} \sigma +k_{1}\cdot \left(\frac{N_{\text{c}}-N_{\text{e}}}{N_{\text{c}}}\right)\cdot e^{\left(-N_{\text{e}}/N_{\text{c}}\right)}, & N_{\text{e}}<N_{\text{c}},\\ \sigma \cdot N\left(0,1\right), & N_{\text{c}}\leq N_{\text{e}}\leq N_{\text{f}},\\ k_{2}\cdot e^{\left(-N_{\text{f}}/N_{\text{e}}\right)}\cdot N\left(0,1\right), & N_{\text{e}}>N_{\text{f}}, \end{array}\right. \end{array}$$

where *k*_1_ and *k*_2_ are regulatory factors in different evaluation process; *σ* represents the noise intensity and *σ* ∈ (0,1); and *N*(0,1) is the standard normal distribution noise. It is clear from equation (1) that the value of *δ*(*N*_e_) can be limited to 0-1 by reasonably choosing the parameters. The curve of cognition noise intensity varies with the number of evaluated individuals (Figure 1).

According to different usage scenarios, the composition of each phase of the cognition noise model is also different. When users are familiar with target products and color images, they may skip the cognition phase; if the number of product color schemes is small and optimized schemes can be gained without too many evaluations, then the fatigue phase will not come.

### 2.2. An Algorithm for Solving Users' Cognition Noise Model

The key to solve users' cognition noise model is to determine the recognition threshold *N*_c_ and the fatigue threshold *N*_f_. The basis for this is the consistency between user's cognition and preference, which means that similar product colors will be given similar judgment. If the condition is met, users are in the intermediate phase; otherwise, they are in the cognition phase or fatigue phase.

An individual *X* can be coded as follows:

(2) $$\begin{array}{c} X=\left\{\left(x_{1},r_{1},g_{1},b_{1}\right),\left(x_{2},r_{2},g_{2},b_{2}\right),\ldots ,\left(x_{m},r_{n},g_{n},b_{n}\right)\right\}, \end{array}$$

where *m* represents partition number of a product for color design; *n* is the number of colors; and *r*_*i*_, *g*_*i*_, and *b*_*i*_ represent RGB parameters of a color within the range of 0–255. Generally, the color number of a product is less than 3, while the number of product form components is more than 3. Therefore, in the color design process, no more than 3 colors are randomly selected and assigned to the product form components.

Considering the area proportion of each color, the similarity of two individuals *X*_*i*_ and *X*_*j*_ can be computed with

(3) $$\begin{array}{c} D_{ij}=1-\overset{m}{\underset{k=1}{\sum }}\frac{{\text{SM}}_{k}}{\text{TA}}\cdot \sqrt{{\left(\frac{r_{ik}-r_{jk}}{s_{r}}\right)}^{2}+{\left(\frac{g_{ik}-g_{jk}}{s_{g}}\right)}^{2}\;+\;{\left(\frac{b_{ik}-b_{jk}}{s_{b}}\right)}^{2}}, \end{array}$$

where *s*_*r*_, *s*_*g*_, and *s*_*b*_ represent standard deviations of *r*, *g*, and *b*, respectively; $s_{r}=\sqrt{1/m\sum_{i=1}^{m}\left(r_{i}-\bar{r}\right)}$ and similarly, we have the value of *s*_*g*_ and *s*_*b*_; SM_*k*_ is the color area of product component *k*; and TA is the total area of a product color scheme.

Due to that users' perception about product color design schemes are emotional and cannot be represented with the precise value, it is necessary to utilize fuzzy numbers to substitute exact values. Triangular and trapezoidal-shaped fuzzy numbers, with bounded interval of [0, 1], are the most widely used to represent uncertainty, and trapezoidal is more general than triangular [40]. Since they provide an intuitive way to capture the vagueness of users' evaluation, we choose trapezoidal fuzzy numbers to denote users' preference of individuals.

For a trapezoidal fuzzy number $\tilde{A}=\left(a,c,d,b\right)$, the membership function can be written as follows:

(4) $$\begin{array}{c} A\left(x\right)=\left\{\begin{array}{cc} \frac{x-a}{c-a}, & a\leq x\leq c,\\ 1, & c\leq x\leq d,\\ \frac{b-x}{b-d}, & d\leq x\leq b,\\ 0, & \text{otherwise}, \end{array}\right. \end{array}$$

where 0 ≤ *a* ≤ *c* ≤ *d* ≤ *b* ≤ 1 and [*a*, *b*] is the support of the fuzzy number and [*c*, *d*] is the modal interval. For ranking design schemes, the defuzzification value of the trapezoidal fuzzy number is needed by using (*a*+*b*+*c*+*d*)/4 [41].

Using a 7-point labeled scale, which is commonly used to gather respondents' ratings for perceptual items [6], users' preference of color images in the IGA can be described by a 4-tuple. Each Kansei attribute comprises 7 sets of semantic terms, and the corresponding fuzzy numbers are indicated in Table 1.

Assume that there are *q* color image indicators for evaluation. The weight of each indicator, represented by *w*_*k*_ (*k*=1,2,…, *q*), is calculated with the AHP method [42]. Let the preference set of *X*_*i*_ given by users be *v*_*i*_={*v*_*i*1_, *v*_*i*2_,…, *v*_*iq*_} and *v*_*ik*_=(*a*_*ik*_, *c*_*ik*_, *d*_*ik*_, *b*_*ik*_), then the synthetical evaluation of product color scheme *xx*_*i*_ in generation *t* can be calculated as follows:

(5) $$\begin{array}{c} f\left(xx_{i},t\right)=\frac{1}{4}\sum \overset{q}{\underset{k=1}{\sum }}w_{k}\left(a_{ik},c_{ik},d_{ik},b_{ik}\right). \end{array}$$

Accordingly, the similarity between two evaluations can be computed as follows:

(6) $$\begin{array}{c} CD_{ij}=1-\frac{1}{q}\overset{q}{\underset{k=1}{\sum }}{\left(\frac{1}{4}\underset{o=a,c,d,b}{\sum }{\left(w_{k}o_{ik}-w_{k}o_{jk}\right)}^{2}\right)}^{1/2}. \end{array}$$

For two similar product color schemes, if the users' two assessments are of high similarity, the cognition noise is considered to be small and users are in the intermediate phase; if the users' two evaluations are of low similarity, the cognition noise is considered to be large and users are in the cognition phase or fatigue phase. For previous *K* (1 ≤ *K* ≤ *j* − 1) products of individual *X*_*j*_, if |*D*_*ij*_ − *CD*_*ij*_| ≤ *δ* (*δ* represents the cognition difference threshold, *i*=*j* − *K*, *j* − *K*+1, &, *j* − 1) can be met, then users are in the intermediate phase and *N*_c_=*j* − *K*_max_ (*K*_max_ is the maximum *K* that met the formula |*D*_*ij*_ − *CD*_*ij*_| ≤ *δ*). When it is confirmed that users are in the intermediate phase, if the previous *K* products of individual *X*_*j*_ meet the formula |*D*_*ij*_ − *CD*_*ij*_| ≤ *δ* (*i*=*j* − *K*, *j* − *K*+1,…, *j* − 1), then users are in the fatigue phase and *N*_f_=*j* − *K*_max_ (*K*_max_ is the maximum *K* that met the formula |*D*_*ij*_ − *CD*_*ij*_| ≤ *δ*). Otherwise, users are in the cognition phase.

### 2.3. Interactive Product Color Design Process

The aim of interactive product color design is to involve users to interact and assess the fitness of individuals for design evolution by means of interactive genetic algorithms to satisfy the objective desired by users. This process includes three parts: designing fitness function, establishing genetic and mutation mechanism, and planning the implementation process of the proposed algorithm.

#### 2.3.1. Fitness Function

In the light of synthetical evaluation computed with formula (5) and considering users' cognition noise, the fitness function can be depicted as follows:

(7) $$\begin{array}{c} F\left(xx_{i},t\right)=\left\{\begin{array}{cc} f\left(xx_{i},t\right), & N_{\text{c}}\leq i\leq N_{\text{f}},\\ \left(1-\delta \left(xx_{i},t\right)\cdot f\left(xx_{i},t\right)\right), & \text{otherwise}. \end{array}\right. \end{array}$$

Formula (7) indicates that when users are in the intermediate phase, their evaluation about product design schemes can accurately reflect their cognition, and the fitness value equals users' evaluation value. Otherwise, the fitness value of individuals should be subtracted by users' evaluation value from the cognition noise.

#### 2.3.2. Crossover and Mutation

Since trapezoidal fuzzy numbers are employed to depict users' preference, the selection criteria should be in accordance with them and set with semantic terms. Here, we assume that the individuals whose synthetic evaluation is equal to or exceeds high Kansei preference will be selected and enter into the next generation, while the individuals below will be eliminated. Parent individuals are selected in the eliminated individuals according to the level of evaluation and to produce offspring populations through crossover and mutation.

Let there be *n* eliminated individuals and numbers of each preference level be *n*(*MH*), *n*(*M*), *n*(*ML*), *n*(*L*), and *n*(*VL*), respectively, *n*(*MH*)+*n*(*M*)+*n*(*ML*)+*n*(*L*)+*n*(*VL*)=*n*. Let the descending order of them be *n*_1_ > *n*_2_ > *n*_3_ > *n*_4_ > *n*_5_, and the probability for each individual to be selected as the parent individual in its preference level can be computed as follows: *n*_1_/[*n* · *n*(*MH*)], *n*_2_/[*n* · *n*(*M*)], *n*_3_/[*n* · *n*(*ML*)], *n*_4_/[*n* · *n*(*L*)], and *n*_5_/[*n* · *n*(*VL*)].

**Figure 2:**
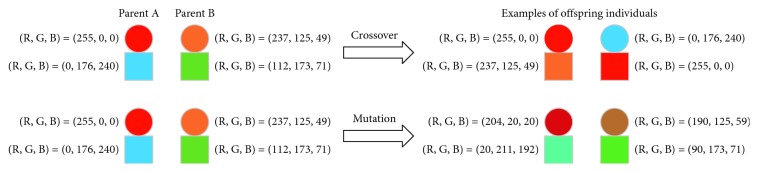
Illustration of crossover and mutation.

For crossover operating, randomly choose the color from parent individuals to make up the required colors of the target product. Mutation is realized by extending R, G, and B values of product color design individuals to 20% with a set mutation rate in each dimension of RGB color space. The changed color values which exceed beyond 0–255 will be ignored.  Figure 2 shows how the crossover and mutation implement.

#### 2.3.3. Interactive Product Color Design Process

The detailed process of the interactive product color design is presented as follows:

1. By specifying the value of *k*_1_, *k*_2_, *σ*, *δ*, and genetic manipulation parameters, the original population of product color schemes is generated
2. The preference of individuals is given by users according to the evaluation indicators of product color images
3. Calculate color value similarity and evaluation similarity of each individual, respectively, in line with formulas (3) and (6)
4. Compute the fitness value of each individual according to formula (7) and save individuals that their evaluation value equals or exceeds specified satisfaction threshold
5. Judge whether the evolutional generation moves outside the set limits. If true, then finish; otherwise, go to next step
6. Judge whether the amount of satisfactory individuals exceeds the set value. If true, then finish; otherwise go to next step
7. Execute crossover and mutation for producing populations of the next generation. And then, go to step 2

**Figure 3:**
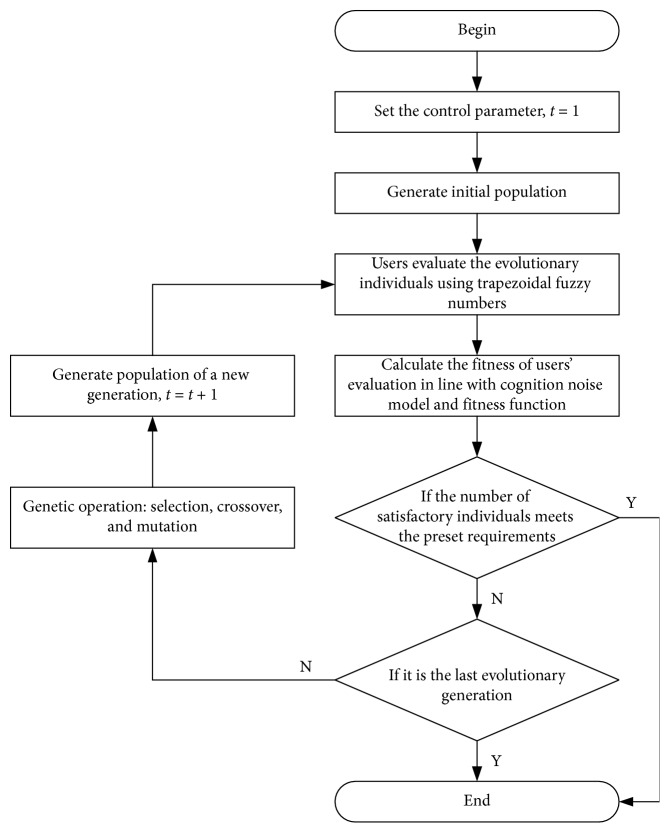
Framework of the proposed method.

The overall framework of the proposed method is shown in Figure 3.

## 3. Case Study

**Figure 4:**
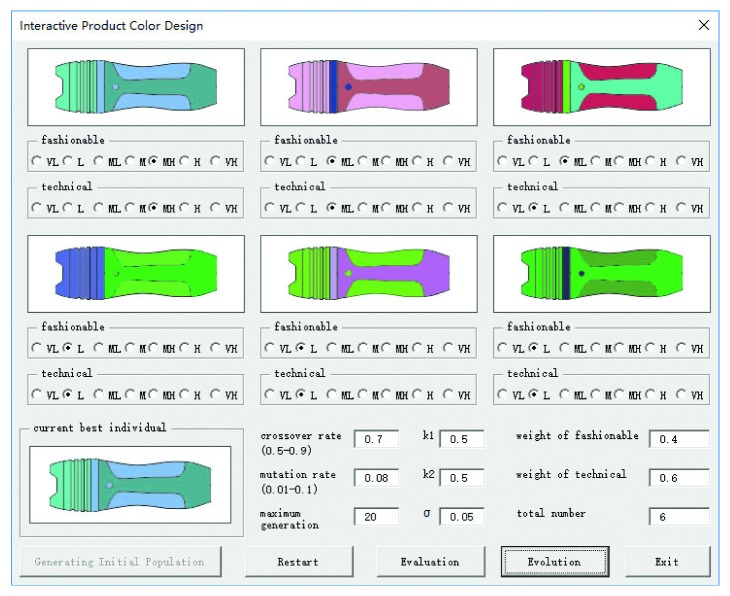
Interactive product color design module.

**Figure 5:**
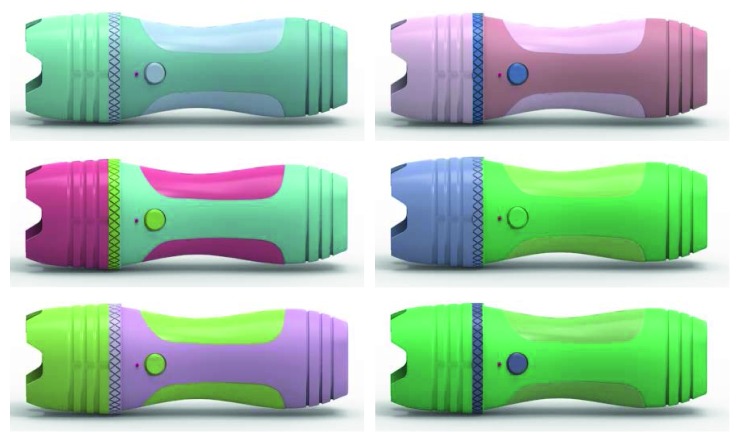
3D configuration and rendering of product color schemes.

The color design of a handheld detector is taken as an example to verify the validity of the proposed method. By utilizing the VBA macroeditor of CorelDRAW software, an interactive product color design module is developed by combining users' cognition noise and an IGA, as shown in Figure 4. There are 6 product color schemes for each generation, and a 7-point labeled scale is deployed to evaluate the indicators which are fashionable and technical. In each generation of evolutionary operations, 3 colors are randomly generated and assigned to 5 product components. In order to better analyze users' perception of product color schemes, we use two computers to implement the experiment. One is used to run the IGA module to quickly generate product color schemes. Another is a workstation where KeyShot software, a real-time 3D rendering software, is installed to give users better visual perception by quickly creating 3D pictures with no more than 30 seconds. 3D configuration and rendering are shown in Figure 5. With the AHP method, by comparing the two indicators, the weights are set by 0.4 and 0.6. The total number of satisfactory solutions required is 6. Through user surveys, individuals whose evaluation equals or exceeds high Kansei preference will be saved as satisfactory solutions. That means the fitness should be greater or equal to 0.8 according to formula (5). Suggested crossover and mutation probability are 0.5–0.9 and 0.01–0.1. Here, we set 0.7 and 0.08, respectively. Maximum evolutionary generation is given 20. Set *k*_1_ = *k*_2_ = 0.5 and *σ* = 0.05. 20 students majored in the industrial design (half male and half female, represented as DM and DF) and 20 students of other majors (half male and half female, represented as CM and CF) are gathered randomly as participants to take part in the experiment. Comparing the proposed method (represented by NIGA) with a traditional IGA, whose parameters of population size, cross probability, mutation probability, and terminate evolutionary generation number are, respectively, set to be 6, 0.7, 0.08 and 20, the calculation results are shown in Tables 2 and 3.

From the experimental results in Table 2, it can be seen that the evaluation of 82.5% users participating in this method has converged, while in the ordinary IGA process, there are 30% participants majored in the industrial design and 35% participants from other majors who have not found the required 6 satisfactory solutions. The convergence rate is increased from 67.5% to 82.5%, which indicates that the proposed method can improve the convergence of the interactive product color design. As shown in Table 2, the cognition threshold indicates that students majored in the industrial design are more familiar with product color image indicators (the mean of *N*_c_ is 2.5), and they can quickly enter the evaluation process and establish mapping between product color schemes and image indicators. While students from other majors need to go through a certain process to digest product color images, and the cognition noise is relatively larger than other students (the mean of *N*_c_ is 5.2). This indicates that knowledge background has a significant impact on users' perception of product color images. In terms of fatigue threshold performance, only 2 industrial design participants did not enter the fatigue phase (for ease of calculation, the number of evaluated individuals after the convergence of evolutionary process was considered to be the fatigue threshold, which are both 96), and all the other participants have got fatigued. The average fatigue threshold is 90.275. For further verification, participants are asked whether they really feel fatigue after the experiment. Investigation shows that 92.1% of them feel confused and cannot judge the image indicators of the color schemes precisely.

From the comparison of average generations shown in Table 3, by contrast with an ordinary IGA, the average generations of NIGA by industrial design students decrease a little (male 1.5 and female 1.8). However, it has a marked impact on students from other majors, with male and female both cutting down 3 generations, which implies that the proposed method plays an active role on users with no or little knowledge of product color design. With the result of improving evolutionary efficiency, the overall average evolutionary generations decrease from 18.15 to 15.825.

In conclusion, as an interactive process involving users into product color design, it is inevitable that users' perception about product color schemes will be influenced by their background knowledge, while cognition noise in different phases will directly affect the validity of product color design. Meanwhile, the precise value used in a traditional IGA cannot represent uncertainty of users' preference. Therefore, integrating users' cognition into product color design process with IGA and trapezoidal fuzzy numbers will be conducive to simulate users' perception about product colors in real world in an objective and scientific way and further improve the convergence speed and evolution efficiency of an ordinary IGA.

## 4. Conclusions

Providing users with multiple product color schemes will help identify users' preference and reduce the risk of product development. Due to cognition difference and uncertainty existing in users, it is not easy to determine which product color users prefer. To assist industrial designers for product color design more effectively and incarnate users' perception about product colors more accurately, interactive genetic algorithms are deployed by combining users' cognition noise with a proposed cognition noise model which consists of three phases: cognition phase, intermediate phase, and fatigue phase. With trapezoidal fuzzy numbers, an algorithm is designed to find key parameters through similarity calculation between RGB values of two individuals and users' evaluations. The interactive product color design process is presented with an instance by comparing with a traditional IGA. By collecting 40 users to participate in the experiment process, the results show that (1) knowledge background will significantly affect users' cognition about product colors; (2) the proposed method is helpful to improve the convergence speed and evolution efficiency with convergence increasing from 67.5% to 82.5% and overall average evolutionary generations decreasing from 18.15 to 15.825.

This study makes the following contributions: (1) Using trapezoidal fuzzy numbers to describe users' preferences makes the application process of an IGA more practical and easy to operate. (2) Incorporating users' subjective cognitive differences into the IGA process will help to improve the convergence speed and evolution efficiency of a traditional IGA. (3) The proposed method can effectively assist industrial designers in the product color design.

## Figures and Tables

**Table 1:** Semantic terms and the corresponding fuzzy numbers for Kansei attribute.

Semantic label	Semantic terms (perceived preference)	Trapezoidal fuzzy number
VL	Very low Kansei preference	(0, 0, 0.1, 0.2)
L	Low Kansei preference	(0.1, 0.2, 0.2, 0.3)
ML	Moderately low Kansei preference	(0.2, 0.3, 0.4, 0.5)
M	Medium Kansei preference	(0.4, 0.5, 0.5, 0.6)
MH	Moderately high Kansei preference	(0.5, 0.6, 0.7, 0.8)
H	High Kansei preference	(0.7, 0.8, 0.8, 0.9)
VH	Very high Kansei preference	(0.8, 0.9, 1, 1)

**Table 2:** Experimental result comparing NIGA with IGA.

Name	User ID	Total generations	*N*_c_	*N*_f_	Total number of satisfactory individuals
NIGA	DM_1_	16	1	91	6
	DM_2_	14	1	80	6
	DM_3_	14	1	82	6
	DM_4_	15	1	87	6
	DM_5_	16	5	None	6
	DM_6_	15	1	85	6
	DM_7_	20	1	115	5
	DM_8_	14	1	79	6
	DM_9_	17	5	100	6
	DM_10_	15	1	88	6
	DF_1_	16	7	91	6
	DF_2_	16	5	None	6
	DF_3_	15	1	89	6
	DF_4_	12	1	68	6
	DF_5_	20	8	103	3
	DF_6_	15	6	88	6
	DF_7_	14	1	79	6
	DF_8_	17	1	100	6
	DF_9_	14	1	80	6
	DF_10_	16	1	92	6

IGA	DM_1_	18	—	—	6
	DM_2_	16	—	—	6
	DM_3_	15	—	—	6
	DM_4_	17	—	—	6
	DM_5_	20	—	—	4
	DM_6_	18	—	—	6
	DM_7_	17	—	—	6
	DM_8_	15	—	—	6
	DM_9_	18	—	—	6
	DM_10_	17	—	—	6
	DF_1_	16	—	—	6
	DF_2_	20	—	—	5
	DF_3_	20	—	—	5
	DF_4_	20	—	—	4
	DF_5_	13	—	—	6
	DF_6_	14	—	—	6
	DF_7_	15	—	—	6
	DF_8_	15	—	—	6
	DF_9_	20	—	—	3
	DF_10_	20	—	—	5

NIGA	CM_1_	13	5	75	6
	CM_2_	14	4	81	6
	CM_3_	15	5	88	6
	CM_4_	20	3	120	3
	CM_5_	15	1	85	6
	CM_6_	20	1	105	5
	CM_7_	14	5	81	6
	CM_8_	15	5	86	6
	CM_9_	15	6	87	6
	CM_10_	20	4	115	5
	CF_1_	15	6	85	6
	CF_2_	12	5	70	6
	CF_3_	14	5	80	6
	CF_4_	20	8	115	6
	CF_5_	15	6	86	6
	CF_6_	16	8	91	6
	CF_7_	14	6	82	6
	CF_8_	20	9	104	4
	CF_9_	15	4	87	6
	CF_10_	20	8	99	5

IGA	CM_1_	19	—	—	6
	CM_2_	20	—	—	6
	CM_3_	19	—	—	6
	CM_4_	20	—	—	6
	CM_5_	20	—	—	4
	CM_6_	18	—	—	6
	CM_7_	17	—	—	6
	CM_8_	20	—	—	5
	CM_9_	20	—	—	5
	CM_10_	18	—	—	6
	CF_1_	18	—	—	6
	CF_2_	19	—	—	6
	CF_3_	20	—	—	5
	CF_4_	20	—	—	5
	CF_5_	18	—	—	6
	CF_6_	20	—	—	4
	CF_7_	19	—	—	6
	CF_8_	20	—	—	4
	CF_9_	18	—	—	6
	CF_10_	19	—	—	6

**Table 3:** Comparison of average generations.

Name	User type	Average generation	Average number of satisfactory individuals
NIGA	DM	15.6	5.9
	DF	15.5	5.7
	CM	16.1	5.5
	CF	16.1	5.7

IGA	DM	17.1	5.8
	DF	17.3	5.2
	CM	19.1	5.6
	CF	19.1	5.4

## Data Availability

The data used to support the findings of this study are included within the article.
